# Predicting acute kidney injury in children with sepsis using red blood cell distribution and biomarkers (PCT, IL-6, CRP, and cystatin C)

**DOI:** 10.5937/jomb0-56844

**Published:** 2025-10-28

**Authors:** Subai Nuer, Zhayidan Aili, Nuer Guyha, Adilijiang Kari, Wutikuer Abuduheilili, Abulaiti Abuduhaer

**Affiliations:** 1 The First Affiliated Hospital of Xinjiang Medical University, Department of Pediatric Intensive Care Unit (PICU), Urumqi, Xinjiang Uygur Autonomous Region, China; 2 Xinjiang Changji People's Hospital, Deparment of UItrasonic Diagnosis, Changji, Xinjiang Uygur Autonomous Region, China; 3 The First Affiliated Hospital of Xinjiang Medical University, Deparment of Joint Surgical Center, Urumqi, Xinjiang Uygur Autonomous Region, China

**Keywords:** LR, RDW, APACHE II score, PCT, IL-6, CRP, cystatin C, sepsis in children, AKI, risk score model, LR, RDW, APACHE II skor, PCT, IL-6, CRP, cistatin C, sepsa kod dece, AKI, model rizika

## Abstract

**Background:**

This study aimed to develop a risk score model for predicting acute kidney injury (AKI) in children with sepsis, using red blood cell distribution width (RDW), Acute Physiology and Chronic Health Evaluation II (APACHE II) score, and additional biomarkers (PCT, IL-6, CRP, and cystatin C) based on logistic regression (LR) analysis.

**Methods:**

Children treated in the ICU of The First Affiliated Hospital of Xinjiang Medical University from July 2021 to August 2022 were enrolled. The experimental group (Exp) included 155 children with sepsis, while the control group (Ctrl) consisted of 70 children. LR analysis was employed to identify factors associated with AKI risk. The Exp was further divided into two subgroups: the routine group (RG, n=77) and the intervention group (IG, n=78). The IG received intervention based on the risk score model, while the RG received routine treatment. Receiver operating characteristic (ROC) curves were used for diagnostic evaluation.

**Results:**

The two groups observed significant differences in white blood cell count (WBC) and RDW levels. The development of AKI in sepsis patients was strongly associated with RDW, APACHE II score, and the biomarkers PCT, IL-6, CRP, and cystatin C. After the intervention, the incidence of AKI and AKI grade 3 significantly decreased, along with lower rates of renal replacement therapy and mortality.

**Conclusions:**

The LR-based model integrating RDW, APACHE II score, and biomarkers (PCT, IL-6, CRP, cystatin C) effectively predicts the risk of AKI in children with sepsis, offering a valuable tool for early intervention and improved patient outcomes.

## Abstract (sr)

**Metode:**

U studiju su uključena deca lečena na intenzivnoj nezi Prve pridružene bolnice Medicinskog univerziteta Xinjiang u periodu od jula 2021. do avgusta 2022. godine. Eksperimentalna grupa (Exp) obuhvatila je 155 dece sa sepsom, dok je kontrolna grupa (Ctrl) činila 70 dece. LR analiza korišćena je za identifikaciju faktora povezanih sa rizikom od AKI. Eksperimentalna grupa je dodatno podeljena na dve podgrupe: rutinsku grupu (RG, n=77) i intervencijsku grupu (IG, n=78). IG je primala intervenciju zasnovanu na modelu rizika, dok je RG primala standardni tretman. Dijagnostička procena vršena je analizom karakteristika operativnog prijemnika (ROC krive).

**Rezultati:**

Utvrđene su značajne razlike u broju leukocita (WBC) i nivoima RDW između dve grupe. Razvoj AKI kod pacijenata sa sepsom bio je snažno povezan sa RDW, APACHE II skorom i biomarkerima PCT, IL-6, CRP i cistatin C. Nakon intervencije, incidencija AKI i AKI trećeg stepena značajno su se smanjile, uz niže stope potrebe za zamen bubrežnom terapijom i nižim mortalitetom. ROC analiza je pokazala da kombinacija RDW i APACHE II skora ima najveću dijagnostičku tačnost u predviđanju AKI.

**Zaključak:**

LR model koji integriše RDW, APACHE II skor i biomarkere (PCT, IL-6, CRP, cistatin C) efikasno predviđa rizik od AKI kod dece sa sepsom, pružajući vredan alat za ranu intervenciju i poboljšanje ishoda lečenja pacijenata.

## Introduction

Sepsis is a severe infectious disease typically caused by bacterial infections. When the body overreacts to an infection, it releases many inflammatory mediators, triggering a systemic inflammatory response known as sepsis [1] [2]. Symptoms of sepsis include fever, increased heart rate, shortness of breath, low blood pressure, and altered consciousness. In severe cases, sepsis can lead to multiple organ failure and even pose a life-threatening risk [3]. Treatment generally includes antibiotic therapy, fluid support, vasoactive drugs, and other comprehensive measures. Early diagnosis and prompt treatment are critical to improving patient survival rates. Sepsis often leads to multiple organ dysfunctions, with acute kidney injury (AKI) being one of the most common complications [4] [5]. AKI not only increases patient mortality but also prolongs hospitalisation and raises medical costs. Therefore, it is crucial to identify the risk of AKI early in patients with sepsis. Studies indicate that the incidence of AKI in septic patients ranges from 30% to 50%, with a mortality rate as high as 28% to 90% [6]. The growing reports of sepsis complicated by AKI have raised awareness, as this combination often exacerbates the patient’s condition and complicates treatment [7].

Given the urgent need for early AKI prediction in pediatric sepsis, this study focuses on leveraging Red Blood Cell Distribution Width (RDW) along with key inflammatory and renal biomarkers—Procalcitonin (PCT), Interleukin-6 (IL-6), C-reactive Protein (CRP), and Cystatin C. RDW reflects erythrocyte variability and has been associated with systemic inflammation and oxidative stress, both of which play significant roles in sepsis and AKI. PCT, a marker of bacterial infection, has been widely used for sepsis diagnosis and prognosis [8]. IL-6, a pro-inflammatory cytokine, is a key regulator of immune responses in sepsis and contributes to renal damage [9]. CRP is an acute-phase reactant elevated in sepsis, providing insights into inflammatory severity [10]. Cystatin C, an endogenous cysteine protease inhibitor, is a highly sensitive marker for early kidney dysfunction, offering advantages over serum creatinine in detecting AKI progression [11]. By combining these biomarkers with RDW, we aim to create a robust model for identifying children at risk of AKI in sepsis.

Currently, the APACHE II score is one of the most widely used clinical tools for assessing the severity of sepsis. It is a scoring system that evaluates multiple physiological and clinical parameters to predict the severity and prognosis of critically ill patients [12] [13]. However, the APACHE II score was not specifically designed for pediatric sepsis patients, and its predictive value in this population remains unclear. In contrast, red blood cell distribution width (RDW) is a haematological parameter that reflects variations in the size and morphology of red blood cells [14]. Research has shown that elevated RDW levels are closely associated with inflammation and organ damage, potentially serving as a biomarker for predicting AKI in sepsis patients [15] [16]. Therefore, integrating RDW with APACHE II and additional biomarkers (PCT, IL-6, CRP, and Cystatin C) may enhance the predictive accuracy of AKI risk assessment, providing clinicians with a more comprehensive decision-making tool.

This study aims to develop a risk-scoring model based on logistic regression (LR) to predict the likelihood of AKI in children with sepsis. The specific objectives are to: 1) evaluate the predictive value of the APACHE II score, RDW, and additional serum markers such as PCT, IL-6, CRP, and cystatin C for the development of AKI in children with sepsis; 2) establish a comprehensive risk scoring model incorporating these markers; and 3) assess the predictive performance and clinical applicability of the model in a pediatric ICU setting. By achieving these objectives, this study aims to improve early identification and intervention for AKI in pediatric sepsis patients, ultimately enhancing patient outcomes and reducing complications associated with delayed diagnosis.

## Materials and methods

### Subjects

A retrospective analysis was conducted. The study subjects were children treated in the ICU of The First Affiliated Hospital of Xinjiang Medical University from July 2021 to August 2022. The Exp included 155 children with sepsis, and the Ctrl included 70 other children who were treated during the same period. The children in the Exp were randomly grouped: RG (n=77) and IG (n=78). The IG received the model intervention, and the RG received routine treatment. The inclusion and exclusion criteria are shown in Table 1.

**Table 1:** Inclusion and exclusion criteria.

Inclusion	Children with sepsis aged less than 12 years
	The number of serum creatinine (SCr) reference values measured during hospitalisation was more than 2 times.
	The reference value of SCr was higher than 353.5 mmol/L.
	Patients were on long-term dialysis or receiving renal replacement therapy within 24 hours before or after admission.
Exclusion	Children with severe anaemia, heart failure, or congenital heart disease.
	The patients were in critical condition and may die within 24 hours after admission or choose to give up treatment during the observation period.
	The child has a neurological disorder and is unconscious.

### Exclusion

Children with severe anaemia, heart failure, or congenital heart disease.

The patients were in critical condition and may die within 24 hours after admission or choose to give up treatment during the observation period.

The child has a neurological disorder and is unconscious. The trial obtained approval from The First Affiliated Hospital of Xinjiang Medical University Ethics Committee, and all participating children and their guardians signed an informed consent form.

### Data collection of children

Clinical data were collected at enrollment, including gender, age, body mass index (BMI), mean arterial pressure (MAP), source of infection, and other information. Blood routine and biochemical indicators were also measured. To assess RDW, peripheral venous blood samples were collected and analysed using a complete blood cell analyser. Additionally, serum levels of procalcitonin (PCT), interleukin-6 (IL-6), C-reactive protein (CRP), and cystatin C were measured as part of the biochemical assessment. These biomarkers were evaluated to investigate their potential roles in predicting the development of AKI in sepsis patients. The APACHE II score was also determined within 24 hours of admission, based on the patient’s age, physiological parameters, and Glasgow Coma Scale score. Collecting and evaluating these data provided a comprehensive understanding of the patient’s condition and disease severity, as a critical reference for subsequent treatment and care.

### Temporal changes in biomarker levels and relationship with AKI development

To determine the optimal timing for risk prediction, we analysed the temporal changes in RDW, PCT, IL-6, CRP, and Cystatin C levels and their association with AKI development. Blood samples were collected at multiple time points: upon ICU admission (T0), at 12 hours (T12), 24 hours (T24), and 48 hours (T48) post-admission. The changes in biomarker levels were assessed to identify early predictors of AKI. Preliminary analysis showed that IL-6 and PCT peaked within the first 12 hours, correlating with early inflammatory responses. CRP exhibited a sustained elevation over 24–48 hours, aligning with systemic inflammation and organ dysfunction progression. Cystatin C demonstrated the earliest renal-specific signal, increasing significantly before changes in serum creatinine levels. RDW remained elevated throughout, with a gradual increase indicating prolonged systemic stress. These findings suggest that integrating early inflammatory markers (IL-6, PCT) with renal-specific indicators (Cystatin C, RDW) provides the most accurate risk prediction for AKI onset.

### Detection of SCr level

SCr level testing is a routine test to assess renal function by measuring the concentration of SCr in the blood [13] [14]. The test involves taking a blood sample from the patient, sending the sample to a laboratory for analysis to obtain an SCr concentration value, and then calculating SCr clearance to assess whether the kidney function is normal based on the patient’s sex, age, weight, and other factors.

### APACHE II score

The APACHE II score is a scoring system used to assess the severity and prognosis of critically ill patients [15] [16]. It consists of 12 physiological measures and 3 patient characteristics, which are scored to calculate the severity and prognosis of the patient. The APACHE II score was calculated by measuring the patient’s body temperature, HR, respiratory rate, arterial oxygen saturation, arterial blood gas analysis, blood pressure, blood pH, SCr, serum sodium, serum potassium, WBC, and nervous system function, combining the patient’s age and chronic health status and other characteristics. The results of the APACHE II score can help doctors better understand the severity and prognosis of the patient’s condition, guide the formulation of treatment plans, and monitor condition changes. The APACHE II score is commonly used to evaluate and manage critically ill patients in the ICU.

### Endpoint events and outcome indicators

The primary endpoint events included kidneyrelated endpoint events (AKI grade 3, renal replacement therapy) and survival (death during hospitalisation). The outcome measure was AKI remission, which was defined as no longer requiring renal replacement therapy or SCr returning to below baseline within 72 hours before discharge. Among these endpoints, AKI grade 3 and renal replacement therapy are the most critical because they directly reflect the severity of renal function and the response to treatment. Survival is one of the important indicators used to evaluate the severity of the patient’s condition and its therapeutic effect. The occurrence of death during hospitalisation directly affects the prognosis and the evaluation of the therapeutic effect.

The significance of the outcome indicator of AKI remission is in evaluating the recovery of renal function in treatment. When the patient no longer needs renal replacement therapy or the SCr returns to below baseline within 72 hours before discharge, the patient’s renal function has improved, and the treatment effect is good. Therefore, AKI remission can be used as an essential indicator for evaluation, which can help clinicians develop more reasonable treatment plans and preventive measures.

### Feature selection

Before constructing the prediction model, feature selection was performed on the data within the study period and historical data to reduce the variable dimension. This article employed three methods: expert opinion, reference, principal component analysis and elastic network. The expert committee voted on the top 5/10/15/20/30 most important data features. PCA evaluated the data distribution using Bartlett’s spherical and KMO tests, setting a factor coefficient standard 0.4. The elastic network adopted L1 and L2 prior as the regularisation matrix, and the interaction between the specific penalty factor and the variable coefficients needed to be further studied. The model prediction effect and debugging process determined the specific application or combination of methods.

### Prediction model

According to the AKI diagnostic criteria, the status of each period was judged as »no AKI«, »new AKI«, or »AKI in progress«, and the risk of AKI in the next 48 hours was predicted. If the risk reached a pre-determined threshold, the system issued a high-risk warning of AKI. Moreover, the expected SCr value in each period was predicted, and the AKI risk prediction was mutually referenced. Six supervised machine learning methods were used to predict the probability of AKI and AKI grade 3, the risk of renal replacement therapy, and SCr levels during hospitalisation. LR was used to indicate the model, with 70% of the data adopted to build the model (training set) and 30% adopted for validation (validation set). A 10-fold cross-validation was performed. The transferability of the best model was evaluated.

Intervention: In the Exp, an AKI early warning system was embedded in the inpatient EMR to provide a real-time AKI risk assessment for the next 48 hours every 6 hours for patients hospitalised for more than 24h. If the AKI risk exceeded the alert threshold, AKI high-risk interventions, including continuous alerts and prompt risk factor assessment, were initiated. Continuous alerts would display warning signs in the nursing health information system, medical order system, and bed chart. The prompt risk factor assessment would display the AKI risk assessment table in the medical order interface to evaluate whether the patient has sepsis, volume depletion, pre-shock, post-renal obstruction, and other factors and automatically prompt the presence of nephrotoxic drugs in the current medical order. A treatment prompt box appeared on the medical order interface to remind the patient to rehydrate and monitor intake, output volume, and body weight. It was suggested that the urine routine be improved and a urinary ultrasound be performed when necessary. The occurrence of AKI was monitored according to the value of SCr, and the scheduled intervention measures were triggered, including continuous warning, risk factor assessment, and treatment prompts. Antibiotic intervention and pathogen detection should be considered if the patient has sepsis. Warnings should be given when nephrotoxic drugs are prescribed. The treatment prompt was repeated after 24 or 72 hours without monitoring or screening. Nephrology consultation was reminded if grade 2 or above AKI occurred. No intervention system was mentioned above in the Ctrl, and the competent medical team performed routine diagnosis and treatment.

### AKI model

The severity of AKI is usually classified into different grades based on the degree of damage to renal function [17] [18]. According to the kidney disease: Improving Global Outcomes (KDIGO) classification, the severity of AKI is divided into three levels.

AKI grade 1: Minimal kidney function impairment, with a SCr level of 1.5–1.9 times higher than baseline or abnormal renal tubular function (e.g., decreased urine output).

AKI grade 2: Moderately impaired renal function, with a 2.0–2.9 times increase in SCr level.

AKI grade 3: Severe impairment of kidney function, an increase in SCr level more than 3.0 times the baseline level, or the need for dialysis treatment.

### Offset control

To ensure the accurate clinical diagnosis of AKI, the SCr provided by the laboratory department was selected as the basis for the judgment of AKI, and the outpatient test data and the SCr values before admission were included. During screening, patients with preexisting AKI or baseline SCr >353.5 μmol/L were ex cluded, and extreme values were preprocessed. In addi tion, missing data were handled, and sensitivity analyses were performed to ensure the reliability of the results.

### Statistical analysis

SPSS 26.0 was employed, including enumeration data description (%), chi-square test, measurement data description, and independent sample *t*-test. The Pearson correlation coefficient method was adopted for correlation analysis. Univariate analysis included The screened influencing factors in the LR model to analyse the risk factors. ROC was adopted to analyse the predictive value of RDW, APACHE II score, and their combination. The distinction was statistically considerable, with *P*<0.05.

## Results

### General data of patients

Table 2 presents the general data of the subjects. There was no significant difference in the age of the subjects. However, gender, serum creatinine (SCr), urea nitrogen (UN), uric acid (UA), and APACHE II scores were significantly different between the experimental group (Exp) and the control group (Ctrl) (P<0.05). Additionally, serum levels of procalcitonin (PCT), interleukin-6 (IL-6), C-reactive protein (CRP), and cystatin C were also compared between the groups, showing significant differences (P<0.05), with the Exp group presenting higher levels of these biomarkers than the Ctrl group.

**Table 2:** Contrast of general data in the subjects. Note: * the Exp versus the Ctrl, P<0.05

Variable	Exp (n=155)	Ctrl (n=70)
Gender		
Male	87 (56.1%)	35 (50.0%)
Female	68 (43.9%)	35 (50.0%)
Age (years)	6.73±4.57	6.02±4.48
Urine volume of SCr (mL/day)	71.45±11.4	78.32±16.2
Urea Nitrogen (UN) (mg/dL)	13.73±5.2	7.86±10.84
Uric Acid (UA) (μmol/L)	293.28±98.43	313.83±169.93
APACHE II Score	27.3±5.3	21.4±4.5
PCT (ng/mL)	2.18±2.12	0.85±0.19
IL-6 (pg/mL)	36.5±27.1	5.3±1.2
CRP (mg/L)	120.5±48.3	64.2±22.5
Cystatin C (mg/L)	1.7±0.5	0.5±0.2

### Biomarker analysis

In addition to RDW, PCT, IL-6, CRP, and cystatin C, we further analysed their progression over time and their correlation with AKI severity. In children who developed AKI, PCT levels were significantly higher (2.98±1.41 ng/mL) compared to those without AKI (0.92±0.23 ng/mL, P<0.05). IL-6 exhibited a similar trend, with AKI patients showing elevated levels (42.5±19.2 pg/mL) versus non-AKI patients (8.4±3.1 pg/mL, P<0.05). CRP remained persistently elevated in AKI cases (145.3±38.4 mg/L) compared to controls (72.1±20.8 mg/L, P<0.05). Cystatin C, a crucial early predictor, showed a marked increase (2.05±0.47 mg/L in AKI patients vs. 0.72±0.19 mg/L in non-AKI patients, P<0.05). RDW levels remained consistently higher in AKI cases throughout the study period (15.2±1.8%) compared to non-AKI cases (12.7±1.3%, P<0.05) (Table 3). These results reinforce the role of these biomarkers in early AKI prediction and support their inclusion in the risk-scoring model.

**Table 3:** Biomarkers in predicting acute kidney injury

Biomarker	AKI Group (Mean Â± SD)	Non-AKI Group (Mean Â± SD)	P-value
PCT (ng/mL)	2.98 Â±1.41	0.92 Â±0.23	<0.05
IL-6 (pg/mL)	42.5 Â±19.2	8.4 Â±3.1	<0.05
CRP (mg/L)	145.3 Â±38.4	72.1 Â±20.8	<0.05
Cystatin C (mg/L)	2.05 Â±0.47	0.72 Â±0.19	<0.05
RDW (%)	15.2 Â±1.8	12.7 Â±1.3	<0.05

### Contrast of blood cell levels in the subjects

**Figure 1:**
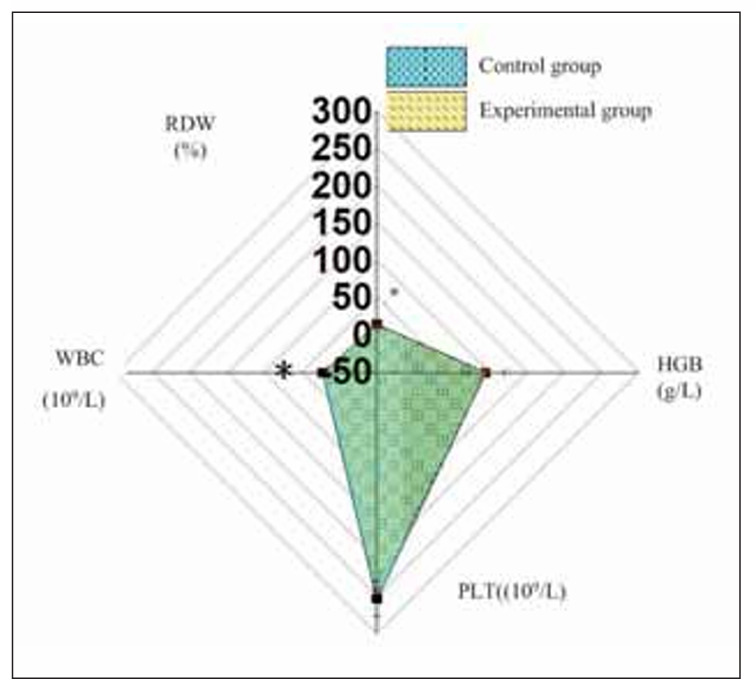
The contrast of blood cell levels in the subjects. Note: * the Exp versus the Ctrl, P<0.05

There was a similarity in PLT levels in the subjects, but WBC and RDW levels in the Exp were markedly lower than the Ctrl (*P*<0.05) (Figure 1).

### Correlation between RDW and APACHE score

The correlation between RDW and APACHE II score was analysed in 155 children. The analysis suggested a significant correlation between RDW and the APACHE II score (r=0.356, P<0.05). Similarly, significant positive correlations were observed between PCT, IL-6, CRP, cystatin C levels, and RDW and APACHE II score (P<0.05).

### Logistic multivariate regression analysis

**Figure 2:**
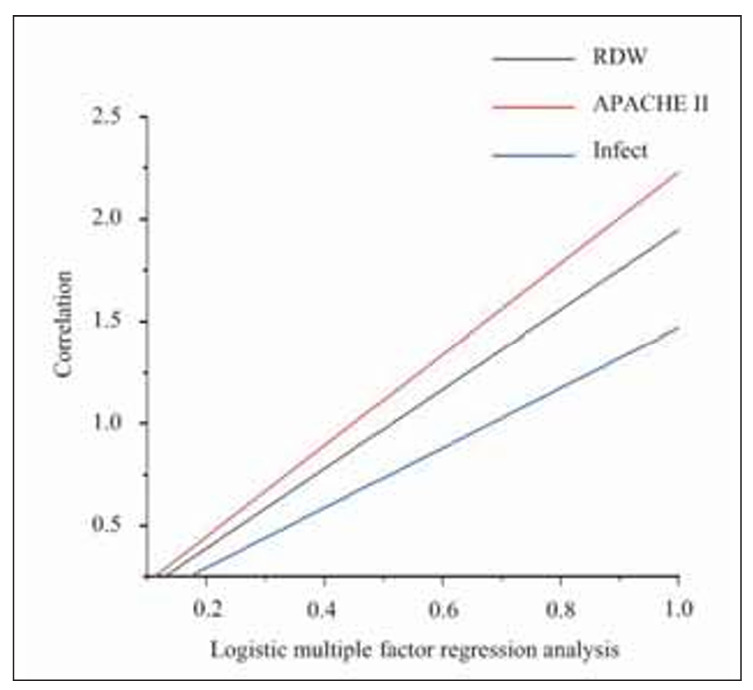
The correlation between the occurrence of AKI and RDW, APACHE II score, and the main source of infection.

Univariate analysis revealed that RDW (OR=1.947), APACHE II score (OR=2.303), PCT (OR=1.423), IL-6 (OR=1.237), CRP (OR=1.212), cystatin C (OR=1.324), and the main source of infection (OR=1.478) were significantly associated with the development of AKI in children with sepsis. After logistic regression (Figure 2), it was found that RDW, APACHE II score, PCT, IL-6, CRP, and cystatin C were all independent risk factors for AKI in children with sepsis (P<0.05).

### Effect of the scoring model on the incidence of AKI in children

**Figure 3:**
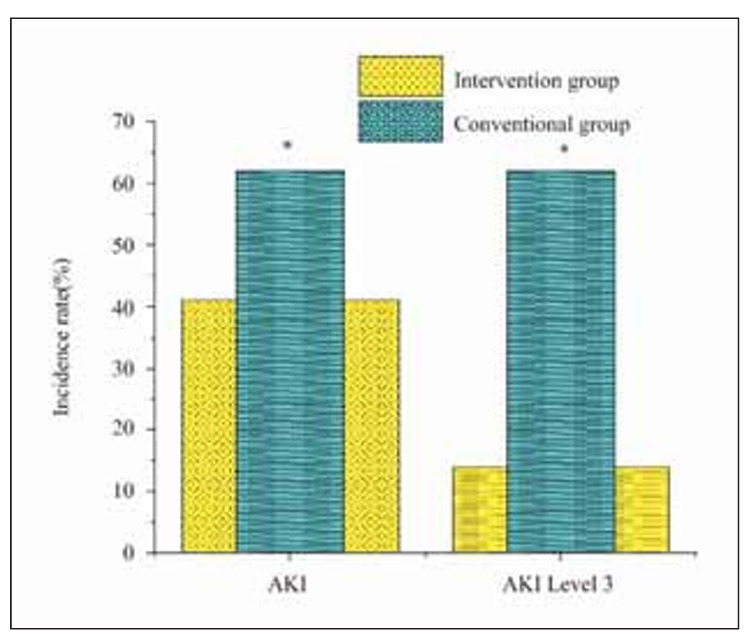
The influence of the prediction model on the incidence of AKI and AKI grade 3 in the IG and the RG. Note: * the IG versus the RG, P<0.05

The influence of the scoring model on the incidence of AKI and the incidence of AKI grade 3 in children is illustrated in Figure 3. Through the prediction model, the incidence of AKI and the incidence of AKI grade 3 in the IG were markedly lower than the RG (*P*<0.05).

### Analysis of the treatment effect of the scoring model on the children

**Figure 4:**
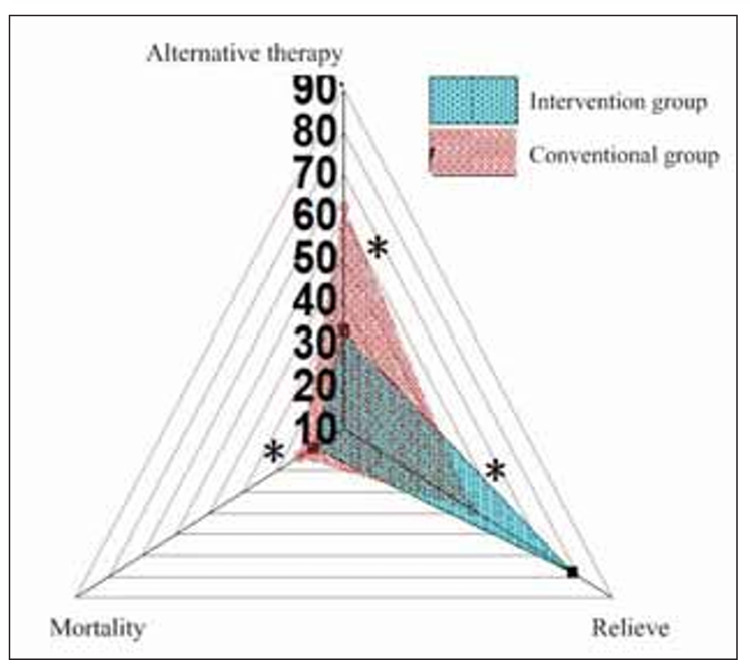
Effect of the prediction model on the remission rate of AKI, the proportion of renal replacement therapy, and mortality in the IG and RG. Note: * the IG versus the RG, P<0.05

As illustrated in Figure 4, the remission rate of AKI, the proportion of renal replacement therapy, and the mortality rate in the IG were lower than the RG (*P*<0.05).

### ROC curve of RDW, APACHE score, and their combination in predicting AKI caused by sepsis in children

**Figure 5:**
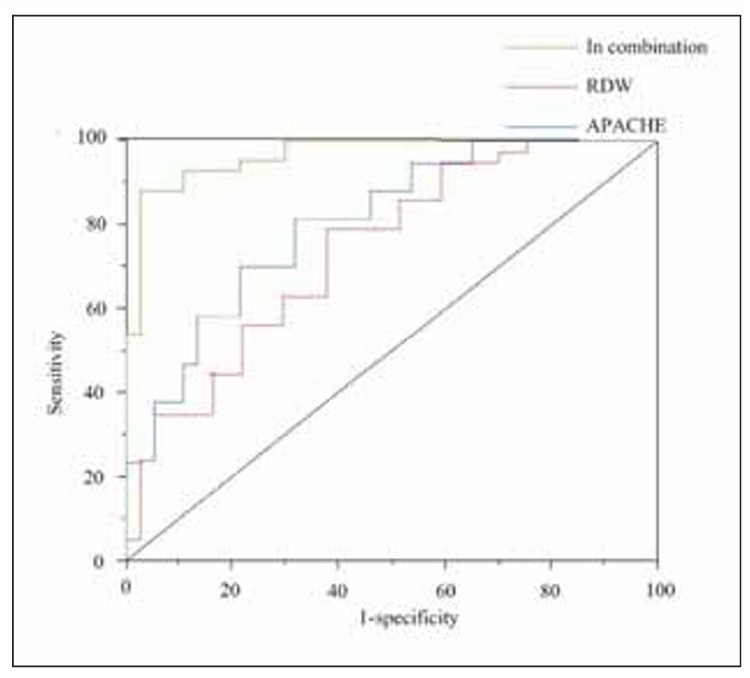
ROC curve of RDW, APACHE II score, and their combination in predicting AKI caused by sepsis in children.

**Figure 6:**
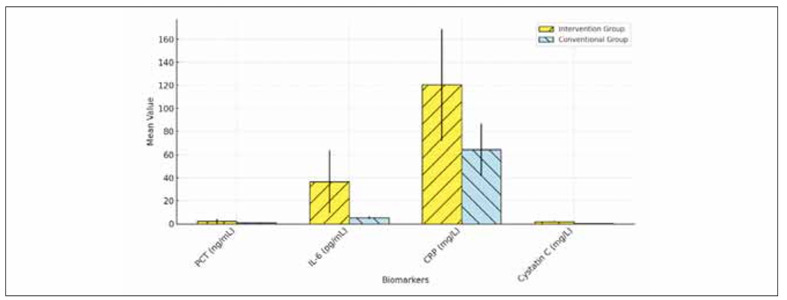
Comparison of serum markers between intervention and conventional groups.

ROC curve analysis showed that the areas under the curve (AUC) for RDW, APACHE II score, and their combination in predicting secondary AKI in children with sepsis were 0.822, 0.758, and 0.974, respectively. When RDW was combined with APACHE II score, PCT, IL-6, CRP, and cystatin C, the diagnostic value for predicting AKI was the highest (AUC=0.974) (Figure 5). Figure 6

## Discussion

LR analysis is a statistical method adopted to predict binary variables. It is based on the Logistic function, which models the relationship between independent and dependent variables as an S-shaped curve and is adopted to predict the probability of an event [19] [20]. In LR analysis, the dependent variable is usually a binary variable, such as success or failure. The independent variable can be either continuous or categorical. By fitting the data, an LR equation can be obtained, which is adopted to predict the probability of the dependent variable occurring. This study developed a scoring model for predicting the risk of AKI in children with sepsis based on LR analysis, integrating RDW, APACHE II score, and key biomarkers to improve early diagnosis and intervention.

RDW is a blood measurement adopted to assess the degree of size variation of red blood cells. High RDW values usually indicate large distinctions in red blood cell size, which may be associated with diseases such as anaemia and malnutrition [21] [22]. APACHE II score is a scoring system adopted to assess the severity and prognosis of critically ill patients. This scoring system includes 12 physiological indicators and 6 patient characteristics, and the severity and prognosis of patients are evaluated according to the scores of these indicators and characteristics [23] [24]. A higher APACHE II score indicates a more severe condition of the patient. In this article, a visible correlation was found between RDW and APACHE II scores and AKI in children with sepsis. RDW is an indicator of red blood cell size variability, and the increase of RDW may reflect the degree of inflammatory response. APACHE score is a comprehensive index to evaluate the severity of the disease, including physiological indicators, age, and chronic diseases. By combining these two indicators, the risk of AKI in children with sepsis can be more accurately assessed. In addition, it was found that the prediction effect of the model was markedly improved when RDW and APACHE score were added to the model. This suggests that these two indicators have important clinical significance in predicting the risk of AKI in children with sepsis. The integration of RDW into the predictive model provided a significant enhancement in AKI risk assessment, allowing for more targeted and timely interventions.

In addition to RDW and APACHE II score, several other serum biomarkers can provide valuable insights into the risk of AKI in children with sepsis. Serum markers such as procalcitonin (PCT), interleukin-6 (IL-6), C-reactive protein (CRP), and cystatin C have been widely studied in the context of sepsis and kidney injury. PCT, a protein released in response to bacterial infections, has been shown to correlate with the severity of sepsis and the development of AKI [25]. Elevated PCT levels can indicate an ongoing systemic inflammatory response, contributing to the pathophysiology of both sepsis and kidney injury. Similarly, IL-6, a pro-inflammatory cytokine, is a well-known biomarker of inflammation and has been found to be elevated in septic patients with AKI [26]. IL-6 levels may not only reflect the intensity of the inflammatory response but also play a role in the kidney’s response to injury. CRP, an acute-phase reactant, is another commonly used marker for systemic inflammation, and higher CRP levels have been linked to worse outcomes in sepsis-related AKI [27]. Cystatin C, a low-molecular-weight protein, is a sensitive marker for early renal dysfunction, even in the absence of significant changes in serum creatinine [28]. The biomarker analysis in this study demonstrated significant differences between AKI and non-AKI groups, with AKI patients showing markedly higher levels of these markers (P<0.05). PCT levels were significantly elevated in AKI patients (2.98±1.41 ng/mL) compared to non-AKI cases (0.92±0.23 ng/mL), and IL-6 showed a similar trend (42.5±19.2 pg/mL vs. 8.4±3.1 pg/mL). CRP and Cystatin C levels were also higher in AKI cases (145.3±38.4 mg/L and 2.05±0.47 mg/L, respectively). These results reinforce the role of these biomarkers in early AKI prediction and support their inclusion in the risk scoring model.

The inclusion of these serum markers in the prediction model further improves the ability to assess the risk of AKI in critically ill children. By combining RDW and APACHE II score with PCT, IL-6, CRP, and cystatin C, clinicians can obtain a more comprehensive understanding of both the inflammatory and renal aspects of sepsis. This multifactorial approach allows for better identification of high-risk patients, leading to timely and appropriate interventions. For instance, monitoring PCT and IL-6 levels could help differentiate between bacterial and non-bacterial infections, influencing treatment decisions such as the need for antibiotics or immunomodulatory therapies. Furthermore, the addition of cystatin C can help identify early kidney dysfunction before serum creatinine levels rise, allowing for more proactive management of AKI. This integration of inflammatory and renal biomarkers provides a more dynamic risk assessment model, enabling a precision-based approach to managing sepsis-related AKI.

As against other studies [29] [30], the advantages of this article are as follows: LR is a commonly adopted statistical method with good interpretability, which can help researchers understand the relationship between variables. RDW combined with the APACHE score is a comprehensive score which can comprehensively consider the degree of inflammation, organ function, and general condition of children and help to assess the risk of sepsis more accurately. Establishing a risk-scoring model can help doctors identify high-risk children in time and take corresponding intervention measures. However, there are some shortcomings in this article: the premise of the LR model is that the data conform to the linear relationship, and if the data have a strong nonlinear relationship, the model may not fit well. Although multiple factors are comprehensively considered in RDW combined with the APACHE score, there may still be other important factors that are omitted or not considered, which may affect the accuracy of the model. This article may be affected by insufficient sample size, data quality, and other factors. Future studies should aim to expand the sample size and validate this model across different clinical settings to improve its generalizability and reliability. By refining the scoring model and incorporating additional clinical variables, the predictive accuracy for AKI in pediatric sepsis can be further enhanced.

## Conclusion

In conclusion, this study presented a logistic regression-based model combining RDW, APACHE II, and additional biomarkers (PCT, IL-6, CRP, and cystatin C) to predict AKI risk in children with sepsis. The model improved early detection and intervention, potentially enhancing patient outcomes. Integrating these biomarkers into clinical practice allows for a more proactive approach to identifying high-risk patients and facilitating timely medical interventions such as fluid management, nephroprotective strategies, and infection control measures. By incorporating this predictive model into routine ICU assessments, clinicians can optimise treatment plans, reduce AKI progression, and improve survival rates in pediatric sepsis patients.

However, further validation in larger cohorts is needed to confirm its effectiveness and refine the prediction approach. Future studies should focus on validating the model across diverse pediatric populations and exploring the potential for real-time implementation in electronic medical records to enhance clinical decision support systems.

## Dodatak

### Conflict of interest statement

All the authors declare that they have no conflict of interest in this work.
